# Prebiotics and Postbiotics Synergistic Delivery Microcapsules from Microfluidics for Treating Colitis

**DOI:** 10.1002/advs.202104089

**Published:** 2022-04-11

**Authors:** Keli Yang, Xiaocheng Wang, Rongkang Huang, Hui Wang, Ping Lan, Yuanjin Zhao

**Affiliations:** ^1^ Department of Colorectal Surgery Guangdong Institute of Gastroenterology Guangdong Provincial Key Laboratory of Colorectal and Pelvic Floor Diseases The Sixth Affiliated Hospital of Sun Yat‐sen University Guangzhou 510655 China; ^2^ Department of Rheumatology and Immunology Nanjing Drum Tower Hospital School of Biological Science and Medical Engineering Southeast University Nanjing 210096 China; ^3^ Biomedical Material Conversion and Evaluation Engineering Technology Research Center of Guangdong Province Institute of Biomedical Innovation and Laboratory of Regenerative Medicine and Biomaterials The Sixth Affiliated Hospital of Sun Yat‐sen University Guangzhou 510655 China; ^4^ Oujiang Laboratory (Zhejiang Lab for Regenerative Medicine Vision and Brain Health) Wenzhou Institute University of Chinese Academy of Sciences Wenzhou Zhejiang 325001 China

**Keywords:** colitis, gut microbiota, microcapsule, microfluidics, postbiotics, prebiotics

## Abstract

Manipulation of gut microbiota by bacterial metabolites has shown protective effects against colitis; while the efficacy is strictly limited by the poor oral delivery efficiency and single drug usage. Here, a novel prebiotics and postbiotics synergistic delivery microcapsule composed of indole‐3‐propionic acid (IPA) postbiotic and three prebiotics including alginate sodium, resistant starch (RS), and chitosan via microfluidic electrospray for preventing and treating colitis are proposed. It is found that oral administration of IPA microcapsules (IPA@MC) to mice can exert significant protective effects to colitis, suggesting the therapeutic synergy between prebiotics and postbiotics. Furthermore, the mechanism of the IPA@MC is revealed in modulating the gut microbiota, that is by significantly increasing the overall richness and abundance of short‐chain fatty acids (SCFA) producing bacteria such as *Faecalibacterium* and *Roseburia*. These results indicate that the prebiotics and postbiotics synergistic delivery microcapsules are ideal candidates for treating colitis.

## Graphical abstract

Novel prebiotics and postbiotics synergistic delivery microcapsule is developed via microfluidic electrospray for treating colitis. Oral administration of the indole‐3‐propionic acid microcapsules can exert significant protective effects to colitis by modulating the health of gut microbiota in mice. The richness and abundance of short‐chain fatty acids producing bacteria are significantly increased after receiving the microcapsule.

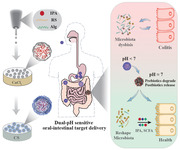

## 1 Introduction

Increasing prevalence of colitis constitutes a major health burden throughout the world due to the unclear multifactorial etiology.^[^ ^1^ ^]^ Generally, the first‐line treatments for colitis are immunosuppressants by suppressing immune responses, which are commonly associated with severe side effects due to their nonspecific immunomodulation.^[^ ^2^ ^]^ As an improvement, the latest treatments such as infliximab and adalimumab have displayed a higher control rate on colitis, but their large‐scale application is severely restricted by the high cost and long‐term use resistance.^[^ ^3^ ^]^ In contrast, recent studies have revealed that the pathogenesis of colitis is closely associated with dysbiosis of gut microbiota.^[^ ^4^ ^]^ Thus, various methods, including probiotics administration and fecal microbiota transplantation, have been proven effective in mitigating colitis by manipulating their gut microbiota.^[^ ^5^ ^]^ Although with some progress, the exogenous gut microbes acquired from these procedures often come with severe side effects, including bloating, abdominal pain and life‐threatening infection.^[^ ^6^ ^]^ Therefore, a safe and effective therapeutic strategy by modulating gut microbiota for controlling colitis is still needed.

**Figure 1:**
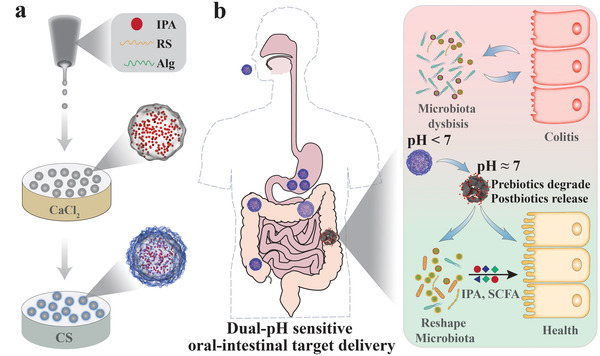
Schematic illustration of the prebiotics encapsulating postbiotic microcapsules (IPA@MC) for preventing and treating colitis. a) The microfluidic electrospray procedures for generating IPA encapsulating Alg/RS microcapsules further coated by chitosan. b) The dual‐pH sensitive core–shell structure allows a slow drug release in the acid condition of the upper gastrointestinal tract and a rapid drug release in the neutral condition of the lower gastrointestinal tract to exert the protective effects against colitis via modulating gut microbiota. Alg: alginate; RS: resistant starch; CS: chitosan; IPA: indole‐3‐propionic acid.

In this study, we aimed to provide a novel prebiotics and postbiotics synergistic delivery microcapsule via microfluidic electrospray for treating colitis, as schemed in **Figure** **1**. Prebiotics are nondigestible dietary carbohydrates including alginate, chitosan, inulin, etc., which have been widely used in diseases with gut microbial dysbiosis and act as drug carriers.^[^ ^7^ ^]^ Apart from prebiotics, postbiotics are the small molecular metabolites digested from prebiotics, which play an important role in intestinal immunomodulatory, epithelium nutrition and microbiota homeostasis.^[^ ^8^ ^]^ Among different postbiotics, tryptophan metabolites including indole‐3‐propionic acid (IPA) and short‐chain fatty acids (SCFAs) are the most attractive candidates for preventing and treating gastrointestinal diseases including colitis, metabolic diseases and cancer.^[^ ^9^ ^]^ A recent study has also revealed these metabolites regulation of gut barriers by regulating the gut microbiota, decreasing inflammation via inhibition of HDAC and activation of HAT in colonocytes.^[^ ^10^ ^]^ However, studies have found low responses to these diseases by oral postbiotics administrations due to the insufficiency of single postbiotics to rebuild a healthy intestine.^[^ ^11^ ^]^ In addition, the oral administration of aqueous postbiotics strictly suffers from poor delivery efficiency to the gastrointestinal tract and limited overall therapeutic efficacy. This evidence suggests that the combined administration of prebiotics and postbiotics can help avoid colitis by increasing the abundance of beneficial bacteria and the level of other postbiotics from the fermentation of prebiotics in the gut. Therefore, a more efficient and controllable carrier for co‐delivering prebiotics and postbiotics is needed in treating colitis. Promisingly, microfluidics has been widely used for the exploitation of intelligent drug delivery systems.^[^ ^12^ ^]^ With the advancement of microfluidic synthesis, drugs with lower molecular weight can be encapsulated into the prebiotics microcapsules, achieving a controllable size distribution and preparation stability. These systems, such as the microfluidic generated microcapsules, can slow down the drug release in the acid upper gastrointestinal tract after oral administration, which enhances the drug concentration in the lower gastrointestinal tract.^[^ ^13^ ^]^ Thus, it is conceived that the intelligent microfluidic microcapsules might provide an efficient venue to co‐delivery prebiotics and postbiotics, as well as offer a new platform for investigating the synergy of prebiotics and postbiotics in treating colitis.

Herein, we employed a capillary microfluidic electrospray technology to generate the desired prebiotic microcapsules with IPA postbiotic encapsulation for the prevention of colitis (Figure 1). The electrostatically driven microfluidics platform ensured the successful encapsulation of IPA into the prebiotic microcapsules composed of alginate and resistant starch (RS), which were further coated by chitosan to prevent IPA leakage in the acid condition. Benefiting from the dual‐pH sensitive core–shell structure, the IPA microcapsules (IPA@MC) showed a slow IPA release in the acid condition of the upper gastrointestinal tract and a rapid IPA release in the neutral condition of the lower gastrointestinal tract. In addition, as the IPA@MC was with a wrinkled surface, they showed a longer retention time in the gastrointestinal tract than the microcapsules with a smooth surface, contributing to the enhanced prebiotics and postbiotics delivery efficacy. Through in vivo experiments, it was found that the oral administration of IPA@MC to mice exerted significant protective effects to colitis than the mice who received prebiotics or postbiotics alone, suggesting that the synergy between prebiotics and postbiotics in IPA@MC offered a more effective therapeutic strategy for colitis. Furthermore, investigations demonstrated that the IPA@MC could modulate the gut microbiome by significantly increasing the overall richness and abundance of SCFA producing bacteria such as *Faecalibacterium* and *Roseburia*. Therefore, such prebiotics and postbiotics synergistic delivery microcapsules are believed to be promising candidates for treating colitis.

## 2 Results and Discussion

**Figure 2:**
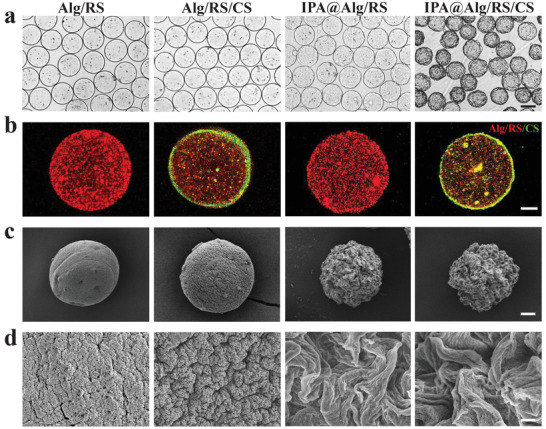
Morphology characterization of the microcapsules. a) Optical, b) fluorescence, and c,d) scanning electron microscope (SEM) images of the Alg/RS, Alg/RS/CS, IPA@Alg/RS, and IPA@Alg/RS/CS microcapsules. The scale bars are a) 200 µm, b) 50 µm, c) 20 µm, and d) 2 µm. Alg: alginate; RS: resistant starch; CS: chitosan; IPA: indole‐3‐propionic acid.

Generally, IPA@MC was prepared via a capillary microfluidic electrospray method followed by coating in a chitosan bath. IPA was homogeneously dispersed in the alginate/RS solution and then transferred to the electrostatically driven capillary microfluidic chips to generate droplets. Next, the droplets were collected in an acidic collection fluid containing 2.0% CaCl_2_. The microcapsules were rapidly solidified by the fast crosslinking between Ca^2+^ and alginate, which firmly restricted the IPA molecules within the alginate/RS networks with little leakage.^[^ ^14^ ^]^ Besides, additional RS mixed with alginate can generate a double‐network with alginate, which can further slow down the release of IPA. Additionally, the IPA@alginate/RS microcapsules were further coated with chitosan via spontaneous electrostatic interactions with alginate to reduce IPA release in the acid condition. As shown in **Figure** **2**a, the obtained IPA microcapsules (IPA@MC) and empty microcapsules (MC) both appeared a highly monodispersed spherical morphology. Confocal fluorescence images further confirmed the core–shell structures of the microcapsules with an Alg/RS core (red) and a chitosan shell (green) (Figure 2b). It was found that the chitosan‐coating microcapsules displayed an obvious smaller diameter than the uncoated microcapsules (Figures S1 and S2, Supporting Information). This phenomenon could be ascribed to the dense macromolecular compounds resulting from the electrostatic interaction between negatively charged amino groups of chitosan and the positively charged carboxyl groups of sodium alginate.^[^ ^15^ ^]^ The spherical shape and size of both chitosan‐coated and uncoated microcapsules could be well controlled by altering the experimental parameters, including the microfluidic flow rate and electrospray voltage (Figure S3, Supporting Information). The microcapsule morphology was further characterized via scanning electron microscopy (SEM) (Figure 2c,d). Interestingly, the IPA@MC exhibited a wrinkled spherical morphology with an extremely rough surface, in remarkable contrast to the smooth surface of the MC without loading IPA. One possible reason for such wrinkled morphology of IPA@MC was that IPA partly precipitated from the acid collection gelling bath, leading to the deformation of Alg/RS spheres. To validate this hypothesis, the wrinkled levels of IPA@MC with different IPA loading amounts were explored, showing a distinct increasing trend with the IPA concentration increased from 1.0% to 3.0% (Figure S4, Supporting Information).

**Figure 3:**
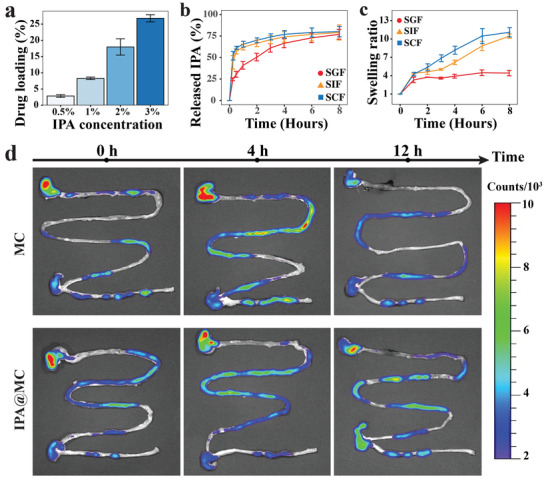
Dual‐pH sensitive drug release and oral‐intestinal target delivery of IPA@MC. a) Drug loading efficiency for IPA@MC with different initial IPA concentrations. b) Cumulative release of IPA from the IPA@MC under different pH conditions including SGF (pH = 1.2), SIF (pH = 6.8), and SCF (pH = 7.4). 2.0% IPA loaded IP@MC were used in the drug release experiment. c) Swelling ratio of the IPA@MC under different pH conditions including SGF (pH = 1.2), SIF (pH = 6.8), and SCF (pH = 7.4). d) IVIS images showing a prolonged retention time of IPA@MC with a wrinkle sphere in the gastrointestinal tract as compared to the MC with a smooth sphere. SGF: simulated gastric fluid; SIF: simulated intestinal fluid; SCF: simulated colonic fluid. MC: empty microcapsules. IPA@MC: IPA microcapsules.

Theoretically, IPA is a small molecular, which would fast escape from the hydrogel networks of microcapsules during the microfluidic electrospray process.^[^ ^16^ ^]^ To reduce IPA leakage during the preparation process, the IPA@ alginate/RS microcapsules were further coated with chitosan. The actual drug loading rate (DLR) of the IPA@MC with initial IPA concentrations of 2.0% was increased from 9.2 ± 2.3% to 18.0 ± 2.5% (w/v) after chitosan coating (Figure S5, Supporting Information). Furthermore, the DLR of IPA@MC increased from 2.86 ± 0.4% to 26.8 ± 1.1% (w/v) with IPA concentration increasing from 0.5% to 3.0% (w/v) (**Figure** **3**a), indicating a positive correlation between the final DLR and initial IPA concentrations. Moreover, the Fourier‐transform infrared (FT‐IR) spectra of IPA@MC revealed the characteristic absorption peaks located around 1700 cm^−1^ for C═O bond and 3440 cm^−1^ for O–H, confirming the successful encapsulation of IPA molecular in the microcapsules (Figure S6, Supporting Information).

In pH‐sensitive drug delivery systems, the transition of pH value is a critical factor to control drug release from the upper to the lower gastrointestinal tract.^[^ ^17^ ^]^ Alginate is a viscous dietary prebiotic consisting of guluronic acid and mannuronic acid that forms a gel at low pH (such as in the stomach), therefore, alginate microcapsule is stable in gastric juice (pH around 1–2), but broken down easily in the intestinal (pH ≥ 7). To validate the dual‐pH sensitive drug release property of the IPA@MC composed of alginate, RS and chitosan, we further examined the drug release and swelling rate of IPA@MC in simulated buffers with different pH values. As shown in Figure 3b, a maximum drug release (>70.0%) of IPA@MC was obtained after 8 h in all buffers. In the acid condition of simulated gastric fluid (SGF), we observed a slow initial release speed of IPA (<50.0%) at the first 2 h, followed by a slow increase of release speed between 2 and 6 h. At last, a cumulative drug release of 75.0% was achieved at 8 h. In contrast, a faster IPA release of 60.0% was observed at the first 2 h in the neutral conditions including simulated intestinal fluid (SIF) and simulated colonic fluid (SCF), and then a cumulative release of 75.0% of drug release was achieved between 4 and 6 h. In line with the drug release pattern, the swelling ratio of IPA@MC immersed in the SIF (10.5 ± 0.4) and SCF (11.1 ± 0.8) was significantly higher than that in the SGF (4.4 ± 0.4) after 8 h (Figure 3c and Figure S7, Supporting Information). All the above results demonstrated the pH‐sensitive drug release capacity of the obtained IPA@MC, which allowed a slower release of IPA and a more stable swelling of IPA@MC in the acid condition of the upper gastrointestinal tract than those in the neutral condition of the lower gastrointestinal tract. As the estimated transition time of food in the upper gastrointestinal tract is around 1–2 h,^[^ ^18^ ^]^ the pH‐sensitive characteristics of the IPA@MC could serve as an excellent drug carrier to deliver postbiotics to the lower gastrointestinal tract by oral administration.

In addition, considering the stronger adhesion ability of the wrinkled microcapsules than the smooth microcapsules, the retention time of IPA@MC with a wrinkled surface could be extended as compared to the smooth MC in the digestive tract, which is expected to improve the drug delivery efficiency of IPA@MC to the gastrointestinal tract. As displayed in Figure 3d, the retention time of the smooth (MC) and wrinkled (IPA@MC) microcapsules in the gastrointestinal tract was real‐timely monitored in mice by an in vivo imaging system (IVIS). After oral administration, most microcapsules were firstly located in the stomach both for the MC group and IPA@MC group. 4 h later, the MC signals located in the small intestine was markedly stronger than the IPA@MC signals, indicating a faster gastric emptying rate of the smooth microcapsules. At 12 h, the IPA@MC group maintained strong fluorescence signals of microcapsules located in the stomach, small intestine and cecum. Comparatively, much weaker fluorescence signals were detected throughout the gastrointestinal tract in the MC group. These results suggested that the wrinkled IPA@MC had a prolonged retention time in the gastrointestinal tract up to 12 h as compared to the MC with a smooth surface after oral administration. Therefore, such prebiotic encapsulating drug carrier would provide a prolonged fermentation time for its prebiotic components and further improve its therapeutic efficacy.

Subsequently, the cytocompatibility of IPA and the drug carriers was tested by culturing with NIH 3T3 cells before in vivo experiments.^[^ ^19^ ^]^ As shown in Figure S8 (Supporting Information), all cells were well alive when cultured with IPA for 2 days with gradient concentrations from 0.05 to 1.0 × 10^−3^ m, indicating the good cytocompatibility of IPA. Additionally, the 0.05 × 10^−3^ m IPA‐treated group exhibited a significant increase in cell proliferation in comparison with the control and other IPA‐treated groups. Therefore, 0.05 × 10^−3^ m IPA was selected as a positive control for following cell experiments. Next, the 3T3 cells were treated with the IPA@MC, MC and 0.05 × 10^−3^ m IPA. As shown in Figure S9a (Supporting Information), we found a normal and healthy morphology of cells in all groups, indicating the low toxicity of the microcapsules. In addition, CCK‐8 assay further confirmed the significantly increased cell proliferation in IPA@MC and IPA groups as compared to the groups without IPA treatment (Figure S9b, Supporting Information). This is consistent with previous studies that tryptophan metabolites can accelerate proliferation and protect cells against radiation toxicity in vitro.^[^ ^9a,c^ ^]^ Therefore, the excellent cytocompatibility property of the IPA@MC endowed them with promising potential in oral administration and colitis treatment.

**Figure 4:**
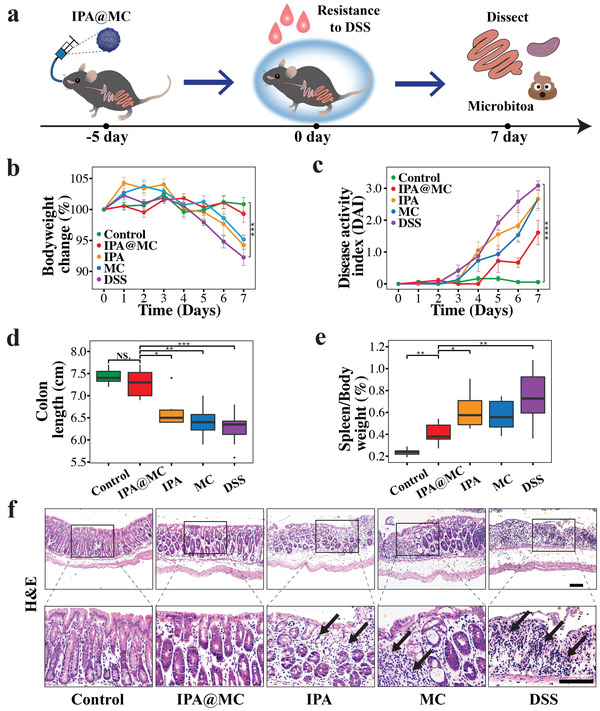
Protective efficacy of IPA@MC against DSS‐colitis in mice (*n* = 6, per group). a) The schematic diagram of animal study design. IPA@MC are expected to exert strong protective effects against DSS‐induced colitis in mice. b) Bodyweight changes from day 0 to day 7. c) Disease activity index (DAI) changes from day 0 to day 7. d) Colon length and e) spleen/body weight ratio of mice on day 7. f) Representative H&E staining images of colon tissues on day 7. The arrows indicate the infiltration of inflammatory cells and epithelial erosions. Student's *t*‐test was applied to test for significance between groups. Error bars indicate SEM. * *p* < 0.05, ** *p* < 0.01, *** *p* < 0.001, **** *p* < 0.0001. NS represent no significance (*p* > 0.05). The scale bar is ) 100 µm in f). Mice were randomized into 5 groups (Control: PBS + normal water; IPA@MC: IPA microcapsules + DSS water; IPA: IPA +DSS water; MC: empty microcapsules + DSS water; DSS: PBS + DSS water).

To explore the protective effect of the IPA@MC against colitis in vivo, IPA@MC, IPA and MC were administrated orally in mice for 5 consecutive days before 7 days colitis induction by dextran sulfate sodium (DSS) in drinking water, as schemed in **Figure** **4**a. Compared with the healthy group, we observed a significant bodyweight loss in the DSS group after DSS treatment. However, there was no significant difference in bodyweight between the mice who received IPA@MC and the healthy mice, indicating that IPA@MC significantly protected the mice against DSS‐induced bodyweight loss (Figure 4b and Figure S10a, Supporting Information). In addition, the IPA@MC group had a significantly lower trend of disease activity index (DAI) than other DSS‐treated groups (Figure 4c and Figure S10b, Supporting Information). Besides bodyweight loss, DSS‐induced colitis is often characterized by a shortening of the colon and an increased ratio of spleen/bodyweight. In our study, the colon length in IPA@MC group was comparable to that in the healthy control group, which is significantly longer than the IPA, MC and DSS groups (*p* < 0.05, Figure 4d and Figure S10c, Supporting Information). Moreover, a significantly lower ratio of spleen/bodyweight in the IPA@MC group was observed as compared to the IPA group and DSS group (*p* < 0.05, Figure 4e), further confirming the colitis‐preventing effect of IPA@MC in mice. The inflammation levels of colonic tissues were examined by hematoxylin–eosin (H&E) and immunohistochemical staining of pro‐inflammatory cytokines including interleukin‐6 (IL‐6) and tumor necrosis factor‐*α* (INF‐*α*). It was found that the H&E staining images did not differ significantly between the healthy control group and the IPA@MC group, in markedly contrast to the severe inflammatory effects as reflected by the loss of crypt structure, epithelial damage and immune cell infiltration in the colon for other DSS‐treated groups (Figure 4f). Furthermore, the expression levels of IL‐6 and INF‐*α* in the IPA@MC group were lower than the IPA, MC, and DSS treatment groups, indicating the ameliorated inflammation by IPA@MC (Figure S11, Supporting Information). Taken together, these results suggested that the IPA@MC exhibited a strong preventive and therapeutic efficacy against the DSS‐induced colitis in mice compared to either IPA or MC alone.

Given that the dietary prebiotics could help modulate gut healthy in colitis, the prebiotics including alginate, RS and chitosan were selected as the carriers for postbiotics in our study. Although both the IPA and MC groups depicted a lower trend of colitis than the DSS group, there was no statistical significance between these groups (Figure 4b,c and Figure S10, Supporting Information), indicating the insufficient therapeutic effects of the single postbiotics or prebiotics. In contrast, the profiles of body weight loss, DAI, colon length and spleen/bodyweight ratio in IPA@MC group all exhibited significant differences as compared to other DSS‐treated groups, suggesting that the synergy between prebiotics and postbiotics in IPA@MC offered a more effective therapeutic strategy for preventing and treating colitis. This result might be attributed to the synergy between prebiotics and postbiotics in the IPA@MC. Prebiotics are mainly nondigestible oligosaccharides that can be utilized by specific beneficial bacteria. As a result, enteric bacteria metabolize these dietary constituents to produce beneficial metabolites (postbiotics) to maintain a healthy gut bacterial flora. On the contrary, it has also been shown that postbiotics are known to increase beneficial bacteria and suppress the growth of harmful bacteria, which further increases prebiotics fermentation in the gut. Then, the increased abundance of beneficial bacteria which fed by prebiotics can produce a higher level of postbiotics to maintain a healthy gut.

**Figure 5:**
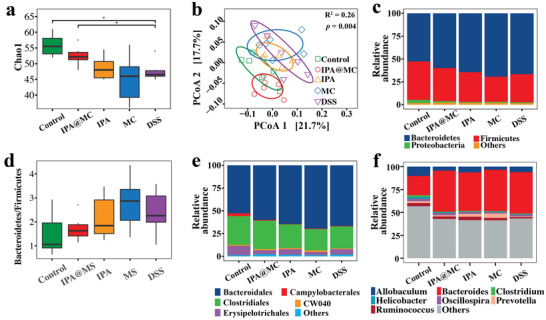
Modulation of gut microbiota using IPA@MC in colitis mice (*n* = 6, per group). a) Chao1 richness among all groups. Student's *t*‐test was applied to test for significance between groups. b) Principal coordinate analysis (PCoA) of Bray–Curtis distance showing the stratification in the gut microbiota of the IPA@MC and the healthy control group from the DSS‐colitis group. Statistical significance was determined by permutational multivariate analysis of variance (PERMANOVA). Relative abundance of the gut bacteria in c) phyla level among all groups. d) Ratio of *Bacteroidetes* and *Firmicutes* level among all groups. Relative abundance of the gut bacteria in e) order and f) genera level among all groups. * *p* < 0.05. Mice were randomized into 5 groups (Control: PBS + normal water; IPA@MC: IPA microcapsules + DSS water; IPA: IPA +DSS water; MC: empty microcapsules + DSS water; DSS: PBS + DSS water).

Emerging evidence suggests the dysbiosis of gut microbiota was closely associated with colitis, including reducing microbiome diversity and the loss of beneficial bacterial taxa.^[^ ^4a^ ^]^ Thus, we examined whether the IPA@MC treatment could modulate the gut microbial profile in DSS‐colitis mice. The 16S rRNA gene sequencing was carried out for the mice fecal samples after 7 days of DSS induction. The IPA@MC treatment significantly increased the gut microbial diversity in colitis mice compared with the IPA, MC, or DSS treatments as estimated by Chao1 richness (**Figure** **5**a). It is worth mentioning that the Chao1 index of IPA@MC group was comparable to the healthy mice. Furthermore, the principal coordinate analysis (PCoA) by Bray–Curtis distance manifested that the mice with IPA@MC treatment had a similar gut microbial profile to the healthy mice, which significantly differed from the mice with IPA, MC, and DSS treatments (*p* = 0.004, Figure 5b). However, the dispersion of observation in the MC and DSS groups has suggested a larger unknown of the complex microbial variations after treatments. Further experiments to enlarge the sample size and use a deeper shotgun metagenomic sequencing to explore the gut microbiome rather than 16s rRNA sequencing might help improve the centroid of the observations within groups.

Subsequently, the gut bacterial composition at phylum, order and genus levels was examined to investigate the taxonomic alterations in colitis mice after various treatments. As shown in Figure 5c, *Bacteroidetes*, *Firmicutes*, and *Proteobacteria* were the predominant bacteria phyla detected in all groups. In particular, the IPA@MC group showed a similar ratio of *Bacteroidetes* to *Firmicutes* to the healthy group with increased abundance of *Firmicutes* and a decreased abundance of *Bacteroidetes*, as compared to the DSS group (Figure 5d). Furthermore, the taxonomic alterations were observed at the order level, including increased *Bacteroidales* and decreased *Campylobacterales*, *Clostridiales*, and *Erysipelotrichales* in the DSS group in contrast to the healthy control group. Consistent with the changes at the phylum level, the mice in IPA@MC group depicted a similar bacterial profile in order level to the healthy mice, while few changes were observed for DSS‐induced mice treated either IPA or MC compared with that in the DSS group (Figure 5e). Next, further alterations of gut bacterial profiles were measured at the genus level (Figure 5f). We only observed modest alterations of the top bacterial genera among groups; this result consisted with previous studies that bacterial taxa with lower abundance might play an important role in the disease occurrence.^[^ ^20^ ^]^

**Figure 6:**
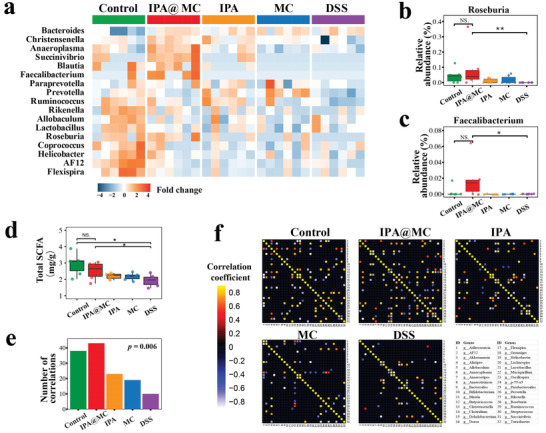
Alterations of gut bacteria and reconstruction of a healthy ecological community by IPA@MC (*n* = 6, per group). a) Altered gut bacterial genera in IPA@MC treated mice depicted a high similarity of gut microbial profile to the healthy mice. b,c) Augmented relative abundance of short‐chain fatty acids (SCFA) producing bacterial genera (*Faecalibacterium* and *Roseburia*) in IPA@MC‐treated mice. Differential taxa were identified by linear discriminant analysis effect size (LEfSe). d) Increased level of total SCFA in cecum samples from IPA@MC treated mice compared to the DSS group mice. Student's *t*‐test was applied to test for significance between groups. e) Number of correlations between gut bacterial genera. Statistical significance was determined by chi‐square test. f) Heatmap showing a higher number of ecological correlations between gut bacterial genera in the IPA@MC group than the DSS group. * *p* < 0.05, ** *p* < 0.01, NS represent no significance (*p* > 0.05). Mice were randomized into 5 groups (Control: PBS + normal water; IPA@MC: IPA microcapsules + DSS water; IPA: IPA + DSS water; MC: empty microcapsules + DSS water; DSS: PBS + DSS water).

The depletion of SCFA producing bacterial taxa was also associated with DSS‐induced colitis and other intestinal diseases.^[^ ^21^ ^]^ We further explored whether the IPA@MC could increase the abundance of postbiotics producing bacteria after oral administration. Thus, the differential gut bacterial genera among all groups were compared by linear discriminant analysis effect size (LEfSe). A total of 17 differential gut bacterial genera were observed in all groups (**Figure** **6**a). Among the differential taxa, the DSS group depicted a decreased relative abundance of SCFA producing bacteria, including *Faecalibacterium and Roseburia*, compared with the healthy mice (Figure 6b,c). Interestingly, the IPA@MC treatment could significantly increase the relative abundance of the SCFA producing bacteria in colitis mice. However, no significant difference in the abundance of these genera was observed between the DSS group and the groups after single IPA or MC treatments. This result confirmed that the synergy between prebiotics and postbiotics provided by the IPA@MC could enhance the restoration of healthy gut microbiota than that of the MC group which only received prebiotics or the IPA group which only received a postbiotic.

In line with the change of abundance of SCFA‐producing bacteria genera after IPA@MC treatment, we found an increased level of total SCFA in the cecum samples from the IPA@MC group as compared to the DSS group (Figure 6d). However, the correlations between the SCFA level and abundance of *Faecalibacterium and Roseburia* failed to reach a statistically significant (Figure S12, Supporting Information). Further studies with large sample size and deeper metagenomics analysis might provide evidence between the SCFA expression and the SCFA‐producing taxa after IPA@MC treatment. Moreover, we analyzed the correlations between these bacterial genera to understand the ecological alterations among all treated groups. It was found that DSS‐induced colitis mice had a fewer number of correlations between bacterial genera than the healthy mice (*p* = 0.006, Figure 6e,f). In comparison, the IPA@MC group presented a higher number of significant correlations than the DSS group, indicating that the IPA@MC was beneficial for the reconstruction of a healthy ecological community for gut microbiota in colitis. All above results suggested that the IPA@MC could reverse the dysbiosis of gut microbiota and thereby exert significant therapeutic efficacy against colitis.

## 3 Conclusion

In summary, we have successfully prepared prebiotic and postbiotic microcapsules, namely IPA@MC, via microfluidic electrospray technology for preventing and treating colitis. Benefitting from the synergy between postbiotic and prebiotic, oral administration of IPA@MC to mice had significant protecting effects against colitis and significantly reshaped the gut microbiota towards the healthy mice. These desirable features render such prebiotics and postbiotics synergistic delivery microcapsules with potential application in preventing or treating colitis.

## 4 Experimental Section

### Reagents

Indole‐3‐propionate acid (IPA) and calcium chloride (CaCl_2_) were purchased from Sigma‐Aldrich. Alginate sodium was purchased from Alta Aesar. Resistant starch (RS) was purchased from Yuanyebio. Chitosan, acetic acid, phosphate‐buffered saline (PBS), sodium chloride (NaCl) and hydrochloric acid were purchased from Aladdin. Nile blue chloride and FITC fluorescence marked polystyrene microspheres (100 nm) were purchased from Dae Technology Co., Ltd. Calcein‐AM and dextran sulfate sodium (DSS) were purchased from Meilunbio. Cell counting kit‐8 (CCK8) was purchased from Dojindo. TNF‐*α* and IL‐6 antibodies for immunohistochemistry were purchased from Servicebio. Mice were provided by Ziyuan Laboratory Animal Technology Co., Ltd. Occult fecal blood assay kit was purchased from Jiancheng Bioengineering Institute.

### Preparation of IPA@MC

The microfluidic chip for microcapsule generation included a capillary (diameter 100–150 µm). IPA (0.5–3.0%, w/v) was homogeneously dispersed in alginate (2.0%, w/v) and RS solution (2.0%, w/v), and then fed in a syringe for microfluidic electrospray. Next, the microfluidic electrospray capillary was held at 8 cm above the surface of the collecting bath with an 8 kV electrostatic potential applied on the microfluidic device to generate droplets. Next, the droplets were collected in an acidic gelling bath containing 2.0% CaCl_2_. The microcapsules were generated by quick crosslinking between Ca^2+^ and alginate. The IPA@MC was solidified in CaCl_2_ bath for 20 min before washing twice with deionized water. After the solidification, 1.0% chitosan was added to the prefabricated IPA@MC with slightly shaking (200 rpm, 20 min) for the formation of a chitosan coating on the microcapsules. At last, the IPA@MC were rinsed twice with deionized water and lyophilized for further use.

### Characterization of IPA@MC

The morphology of IPA@MC was visualized by scanning electron microscopy (SEM, Hitachi) and optical microscopy (OLYMPUS). To visualize the core–shell structure of the microcapsules, the red and green fluorescence‐labeled polystyrene microspheres (2.0%, v/v) were added into IPA/Alg/RS solution and chitosan solution, respectively. Then, the fluorescence images were obtained using a fluorescence confocal microscope (Nikon).

### Drug Loading Rate and In Vitro Drug Release

20 mg of freeze‐dried IPA@MC was immersed in 5 mL of PBS (pH = 7.4) with oscillation (400 rpm, 37 °C, 48 h). Then 200 *μ*L of supernatant was retrieved to measure the IPA concentration. The drug loading rate (DLR) was calculated by the following equation

(1) $$\begin{array}{ccc} \mathrm{DLR}\% & = & \left(\mathrm{amount}\;\mathrm{of}\;\mathrm{drug}\;\mathrm{loaded}\right)/\left(\mathrm{amount}\;\mathrm{of}\;\mathrm{IPA}@\mathrm{MS}\right)\\ & & \times \;100\% \end{array}$$

To simulate the control drug release from the IPA@MC under different pH condition, simulated gastrointestinal fluids were prepared. The simulated gastric fluid (SGF) included 0.09 mol L^−1^ NaCl (pH adjusted to 1.2 by HCl). The simulated intestinal fluid (SIF) and simulated colonic fluid (SCF) were composed of 0.01 m KH_2_PO_4_ and K_2_HPO_4_ (pH adjusted to 6.8 and 7.4 respectively). 20 mg of freeze‐dried IPA@MC was immersed in either 2 mL of SGF, SIF or SCF with oscillation (200 rpm, 37 °C). For each sampling time point, 200 *μ*L supernatant was retrieved for further measurement and 200 *μ*L fresh buffer was added back to the collection tubes. The drug release rate was calculated using the following formula

(2) $$\mathrm{Drug}\;\mathrm{release}\;\mathrm{rate}=\frac{\sum \left(\mathrm{Cn}-1:C1\right)\times V/10\;+\;\mathrm{Cn}\times V}{m_{\mathrm{drug}}}\times 100\%$$

where V is the total volume, *C* is the concentration, *n* is sampling time point and m_drug_ is the drug loading weight.

As shown in Figure S13 (Supporting Information), The absorptance of IPA solutions was measured at 273 nm by a Varioskan LUX multimode microplate reader (ThermoFisher). Next, The concentrations of IPA were calculated by the UV absorptance standard curve for IPA concentrations ranging from 1.95 × 10^−4^% to 6.25 × 10^−4^% (w/v).

### Swelling Rate of IPA@MC

A total of 100 mg freeze‐dried IPA@MC was weighted and allowed swelling at 37 °C in SGF, SIF, and SCF, respectively. After the removal of the extra fluid by filter papers, the swollen IPA@MC was weighed immediately at the designed time points. The equation for the swelling ratio of the IPA@MC was as follows

(3) $$\mathrm{Swelling}\;\mathrm{ratio}=\frac{W_{n}-W_{i}}{W_{i}}$$

where *W_n_* represents the weight of swollen IPA@MC at the time point *n*. *W*_i_ represents the initial weight of the IPA@MC.

### Characterization of IPA@MC Retention Time in the Gastrointestinal Tract

To track the transit of microcapsules through the gastrointestinal tract, Nile blue chloride labeled polystyrene microspheres (5.0%, v/v) were added into IPA/Alg/RS solution to generate fluorescence‐labeled IPA@MC. Each mouse was received ≈200 µL IPA@MC with a wrinkled surface or MC with a smooth surface by gavage, then sacrificed at 0, 4, and 12 h after the oral administration. The transit of IPA@MC in the gastrointestinal tract was visualized by an in vivo imaging platform (IVIS, PerkinElmer).

### Cytocompatibility of the IPA@MC

The cytocompatibility was evaluated by coculturing IPA@MC with 3T3 cells for 3 days. Cells were treated with IPA@MC (500 µg mL^−1^), MC (500 µg mL^−1^), and IPA (0.05 mmol L^−1^) solutions, respectively. Cells without treatments were used as a control. Cell Counting Kit‐8 (CCK8) tests were performed to measure cell proliferation. Live or dead cells were visualized by Calcein‐AM/Propidium Iodide staining. The fluorescence images were taken by an inverted fluorescence microscope (Zeiss).

### Animal Experiment

Male C57BL/6J mice aged 6–8 weeks were randomized into 5 groups (*n* = 6 per group) : 1) IPA@MC group: DSS + IPA@MC (2.5 mg/10 g bodyweight per day), 2) MC group: DSS + MC (2.5 mg/10 g bodyweight per day), 3) IPA group: DSS + IPA solutions (0.5 mg/10 g bodyweight per day), 4) DSS group: DSS + PBS (0.2 ml/20 g bodyweight per day), and 5) Control group: PBS (0.1 mL/10 g bodyweight per day). To reduce the heterogeneity of the gut microbiota, all the mice were cohoused long to 7 days before randomized into different treatment groups. The mice in colitis groups were provided DSS (3.0%) in drinking water ad libitum to induce colitis for 7 days. All the mice received oral gavage once per day from 5 days before initial DSS treatment and stopped at 7 days after initial DSS treatment. Bodyweight and DAI were collected daily to monitor diseases severity. Colon length, spleen weight, feces, and colon tissues were collected on day 7. The approval for the animal experiment was acquired from the Animal Investigation Ethics Committee of The Sixth Affiliated Hospital of Sun Yat‐sen University.

### Histology Analysis and Immunofluorescence Analysis

The distal colonic samples were fixed in paraformaldehyde solution (4.0%) for further histological analysis. Hematoxylin and eosin (H&E) staining, INF‐*α* immunohistochemical staining and IL‐6 immunohistochemical staining were conducted to assess the inflammatory level. Histological images were taken using optical microscopy (Nikon). All the histologic scoring was performed by double‐blinded assessments.

### 16S rRNA Gene Sequencing for Gut Microbiota

Approximately 100 mg of mice feces were used for extracted DNA by E.Z.N.A. Soil DNA Kit following the manual instruction. The DNA concentration was quantified by NanoDrop2000 (Thermo Fisher Scientific) and the nucleic acid integrity was measured by electrophoresis. The V_3_‐_4_ hypervariable area of 16S rRNA gene was amplified by primers (341F and 806R). Next, DNA libraries were constructed through end repairing, purification, and PCR amplification. Illumina Novaseq 6000 platform was used to generate sequencing reads with pair‐end 250 strategy by Wekemo Technology Co., Ltd. (Shenzhen, China).

### Gut Microbiota Analysis

The raw sequencing reads were demultiplexed and adapter trimmed by QIIME (v1.7.0), then imported to QIIME2 (v2021.4) for downstream analysis.^[^ ^22^ ^]^ Briefly, quality control was performed by default parameters; then the DADA2 plugin was used to extract the amplicon sequence variant (ASV) table. Furthermore, taxonomic annotation was assigned by reference to Greengenes database (v13.8).

### Measurement of SCFA in Cecal Contents

Approximately 50 mg of cecal contents were used for the measurement of SCFA composition including acetic acid, propionic acid, isobutyric acid, butyric acid, isovaleric acid, valeric acid and caproic acid. The concentration of SCFA was quantified using gas chromatography–mass spectrometry (GC‐MS) with an Agilent HP‐INNOWAX platform (7700A).

### Statistical Analysis

Statistical comparisons between groups were performed by analysis of variance (ANOVA) test and Student's *t*‐test for continuous variables. For continuous variables, mean (SD) are presented unless noted in the figure legend. Distributions between groups were compared by chi‐squared test. LEfSe analysis was performed to identify differential gut microbial taxa. The relative abundance table for gut microbial profiles was transformed from the reads count table after subsampling the reads to equal number per sample. Correlation between two continuous variables was calculated by Pearson correlation. SparCC was performed to identify the correlation coefficient between taxa. All statistical analyses and visualization were processed by the R project (v3.61, Vienna, Austria).^[^ ^23^ ^]^ *p* values less than 0.05 were regarded as statistically significant. All tests were performed with two‐sided testing with alpha of 0.05.

## Conflict of Interest

The authors declare no conflict of interest.

## Author Contributions

K.L.Y. and X.C.W. contributed equally to this work. Y.J.Z. conceived the idea and designed the experiment; K.L.Y. conducted experiments and data analysis; K.L.Y., X.C.W., R.K.H., H.W., P.L., and Y.J.Z. wrote the manuscript.

## Supporting information

Supplementary Information

## Data Availability

The data that support the findings of this study are available from the corresponding author upon reasonable request.
