# Supplementary material for: Prebiotics and Postbiotics Synergistic Delivery Microcapsules from Microfluidics for Treating Colitis
Source: Adv Sci (Weinh). 2022 Apr 11;9(16):2104089. doi: 10.1002/advs.202104089 (PMC9165482; doi:10.1002/advs.202104089)
Supplement: Supplementary file 1 — Supplementary Information [file ADVS-9-2104089-s001.pdf]

Supplementary

Prebiotics and Postbiotics Synergistic Delivery Microcapsules from Microfluidics for Treating Colitis

Keli Yang[1,2,3], Xiaocheng Wang[2,4], Rongkang Huang[1,3,], Hui Wang[1,3,*], Ping Lan[1,3,*], Yuanjin Zhao[2,4,*]*

1. Department of Colorectal Surgery, Guangdong Institute of Gastroenterology, Guangdong Provincial Key Laboratory of Colorectal and Pelvic Floor Diseases, The Sixth Affiliated Hospital of Sun Yat-sen University, Guangzhou 510655, China
2. Department of Rheumatology and Immunology, Nanjing Drum Tower Hospital, School of Biological Science and Medical Engineering, Southeast University, Nanjing 210096, China
3. Biomedical Material Conversion and Evaluation Engineering Technology Research Center of Guangdong Province; Institute of Biomedical Innovation and Laboratory of Regenerative Medicine and Biomaterials, The Sixth Affiliated Hospital of Sun Yat-sen University, Guangzhou 510655, China
4. Oujiang Laboratory (Zhejiang Lab for Regenerative Medicine, Vision and Brain Health), Wenzhou Institute, University of Chinese Academy of Sciences, Wenzhou, Zhejiang 325001, China

Co-Correspondence*:

Yuanjin Zhao, Professor, yjzhao@seu.edu.cn

Ping Lan, Professor, lanping@mail.sysu.edu.cn

Hui Wang, Professor, wang89@mail.sysu.edu.cn

Rongkang Huang, Professor, huangrk3@mail.sysu.edu.cn

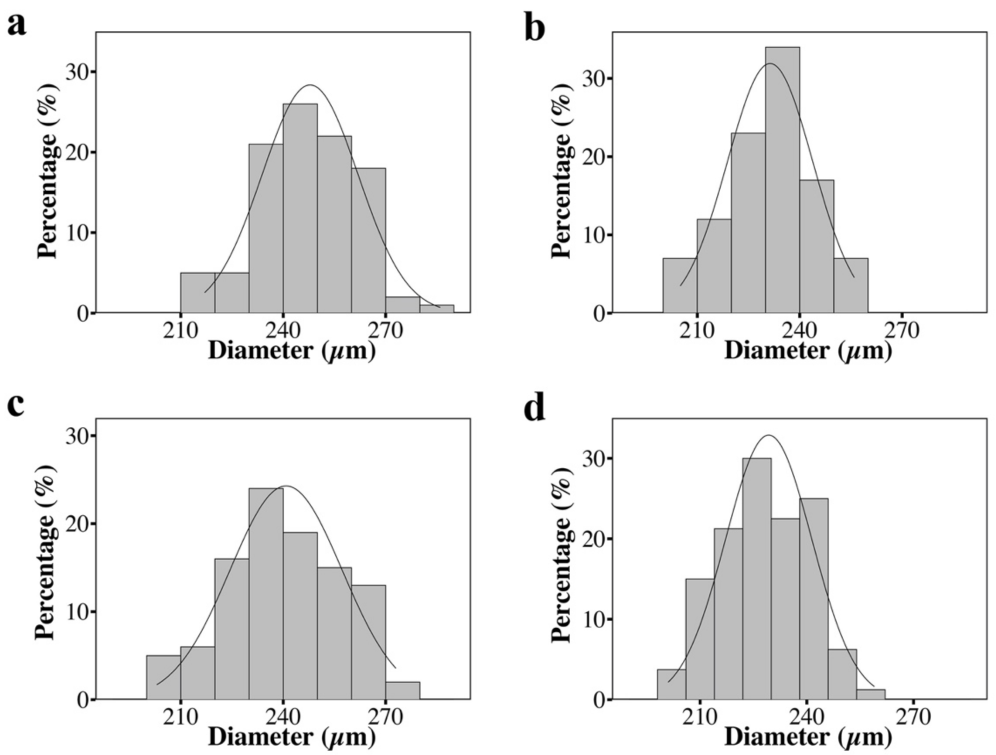

Figure S1. Diameter distribution of the (a) Alg/RS, (b) Alg/RS/CS, (c) IPA@Alg/RS and (d) IPA@Alg/RS/CS microcapsules. Alg: alginate. RS: resistant starch. CS: chitosan. IPA: indole-3-propionic acid.

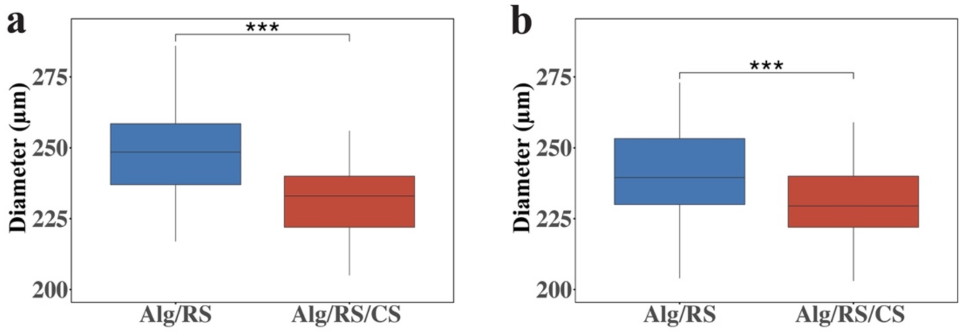

Figure S2. Decreased diameter of (a) microcapsules (MC) and (b) IPA microcapsules (IPA@MC) after coating chitosan. Student's t-test was applied to test for significance between groups. *** $p < 0.001$.

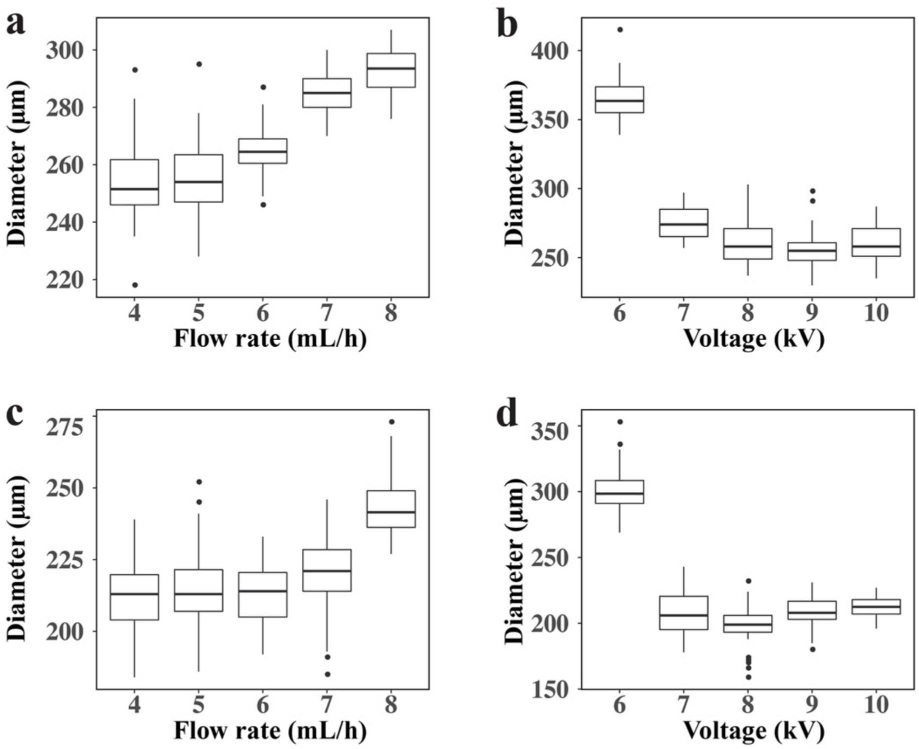

Figure S3. The influences of the (a, c) flow rate and (b, d) voltage on the sphere diameter of IPA@MC before (a, b) and after chitosan coating (c, d).

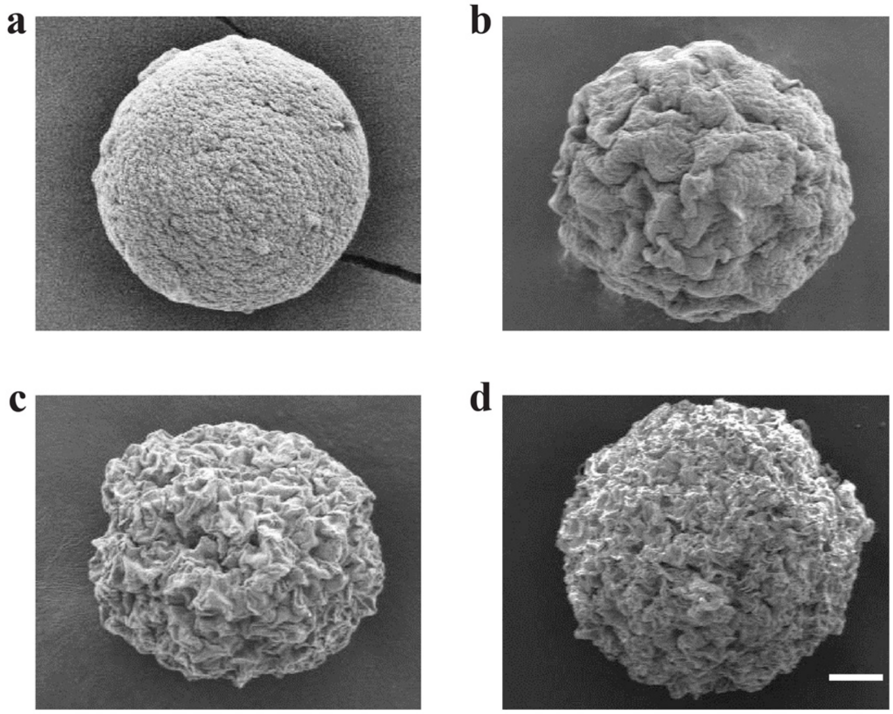

Figure S4. SEM images showing increased wrinkling level of the IPA@MC surface with increasing IPA concentrations of (a) 0%, (b) 1%, (c) 2% and (d) 3%. The scale bar is 20 μm.

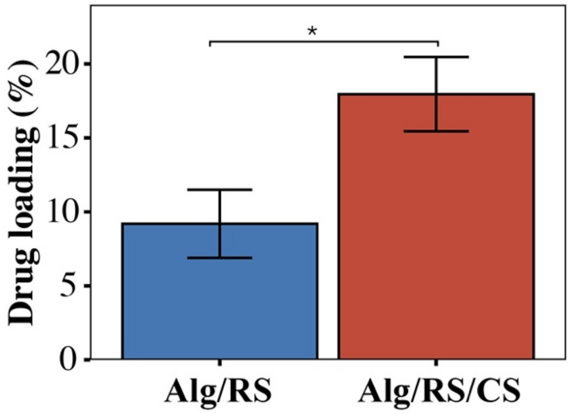

Figure S5. Increased DLR for IPA@MC after chitosan coating compared with IPA@MC without chitosan coating. IPA@MC with initial IPA concentrations of 2.0% was measured between groups. Student's t-test was applied to test for significance between groups. * $p < 0.05$.

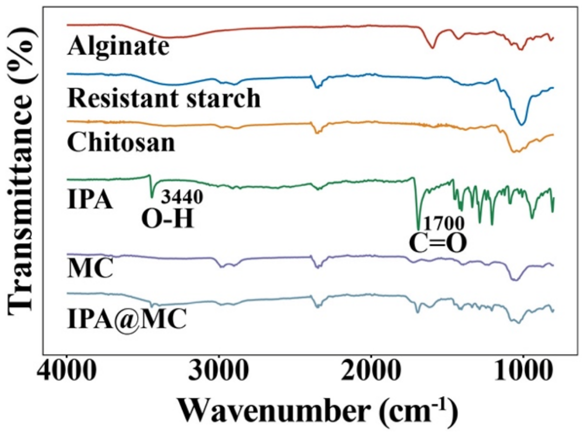

Figure S6. FTIR spectra of IPA@MC and its gradients.

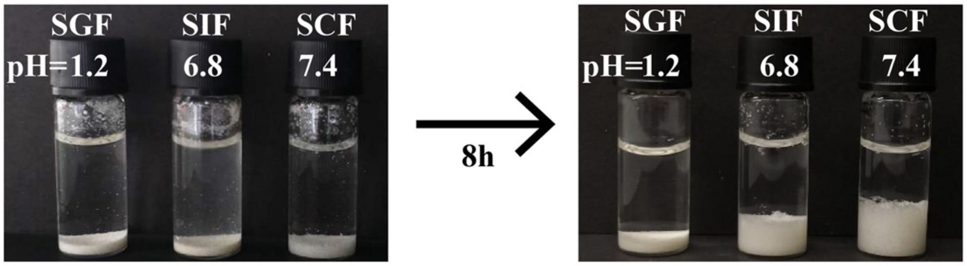

Figure S7. Digital photos of the swelling IPA@MC after 8 hours in SGF (pH = 1.2), SIF (pH = 6.8) and SCF (pH = 7.4). SGF: simulated gastric fluid; SIF: simulated intestinal fluid; SCF: simulated colonic fluid.

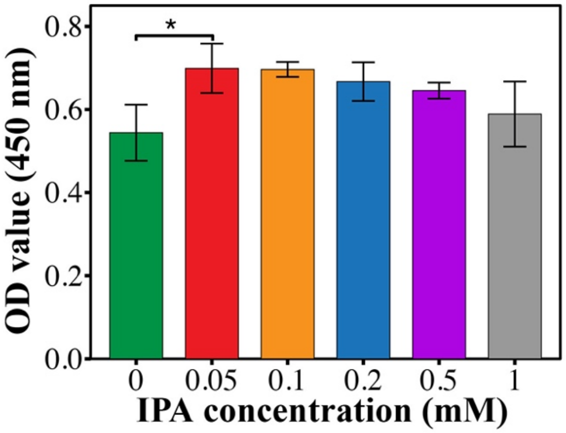

Figure S8. Proliferation of 3T3 cells treated with different concentrations of IPA from 0 to 1 mM for 2 days. The cell proliferation was measured by CCK-8 assay. The OD value was measured at 450 nm. Student's t-test was applied to test for significance between groups. * $p < 0.05$.

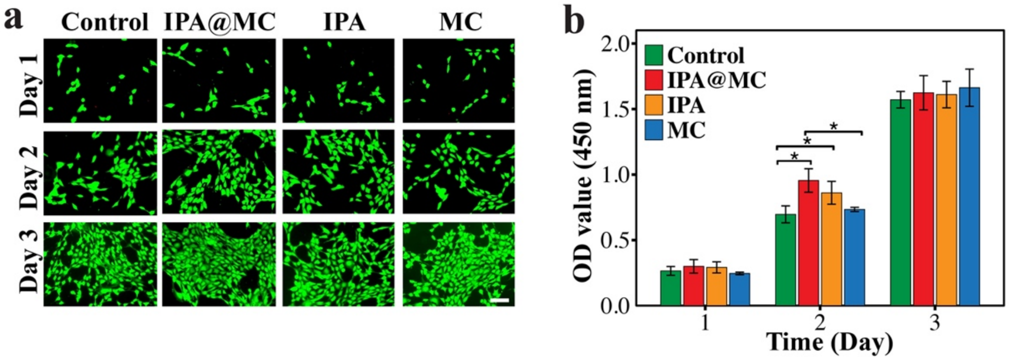

Figure S9. Cytocompatibility of IPA microcapsules. (a) The fluorescent images of 3T3 cells co-culture with IPA@MC, 0.05 mM IPA and MC. (b) Proliferation of 3T3 cells measured by CCK-8 assay from day 1 to day 3 . The OD value was measured at 450 nm. The scale bar is 100 μm in (a). Student's t-test was applied to test for significance between groups. * $p < 0.05$.

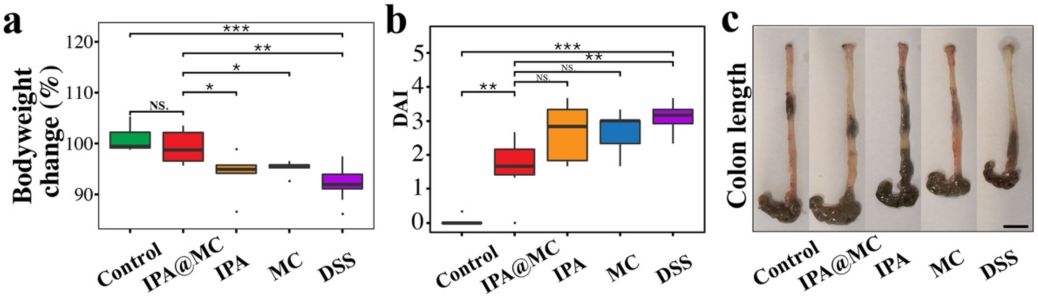

Figure S10. Protective efficacy of IPA@MC against DSS-colitis in mice on day 7 (n=6, per group). Body weight change (a), disease activity index (b) and colon length (c) for the mice after various treatments. Student's t-test was applied to test for significance between groups. ** $p < 0.01$, *** $p < 0.001$. DAI: disease activity index. The scale bar is 1 cm in (c). Mice were randomized into 5 groups (Control: PBS + normal water; IPA@MC: IPA microcapsules + DSS water; IPA: IPA + DSS water; MC: empty microcapsules + DSS water; DSS: PBS + DSS water).

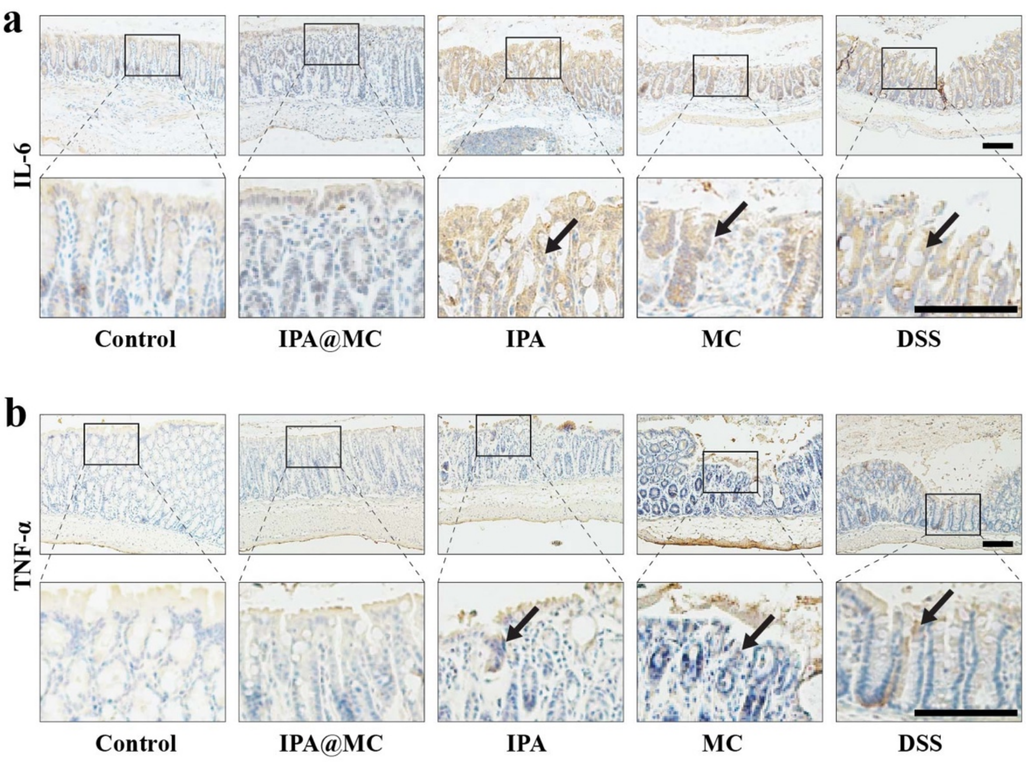

Figure S11. Representative IHC staining images for IL-6 (a) and TNF-α (b) on day 7. The scale bar is 100 μm. The arrows indicate the expression of markers. Mice were randomized into 5 groups (Control: PBS + normal water; IPA@MC: IPA microcapsules + DSS water; IPA: IPA + DSS water; MC: empty microcapsules + DSS water; DSS: PBS + DSS water).

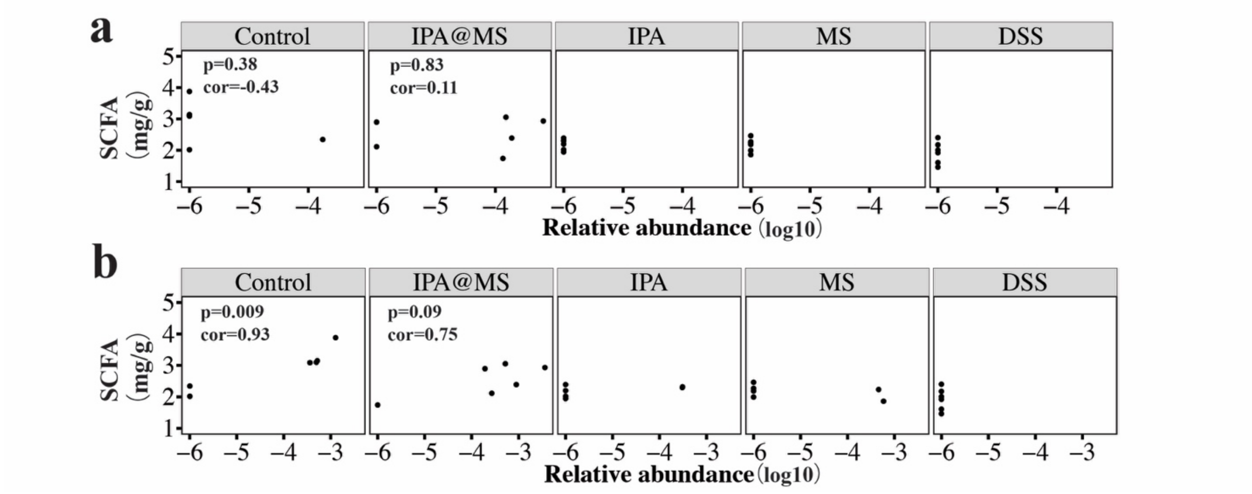

Figure S12. Correlations between total level of SCFA and bacterial genera (a) *Faecalibacterium* and (b) *Roseburia*, respectively. Correlation was calculated by Pearson correlation. Mice were randomized into 5 groups (Control: PBS + normal water; IPA@MC: IPA microcapsules + DSS water; IPA: IPA + DSS water; MC: empty microcapsules + DSS water; DSS: PBS + DSS water).

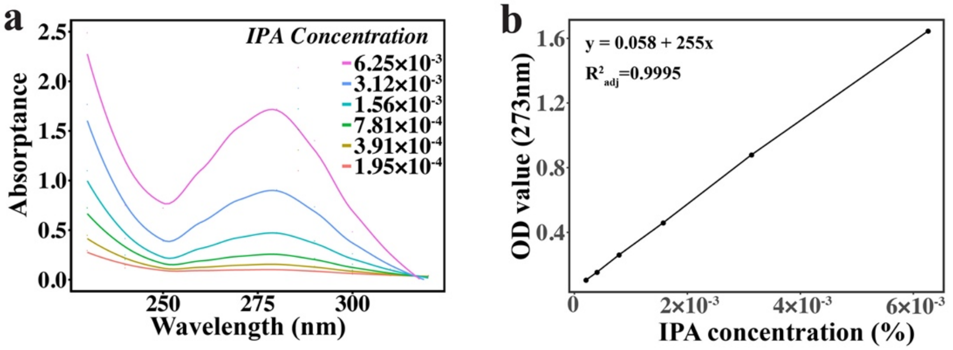

Figure S13. (a) UV absorptance spectra of the aqueous solution with different IPA concentrations ranging from 1.95×10^{-4} % to 6.25×10^{-4} % (w/v). (b) The standard curve indicating the linear correlation between IPA concentration and UV absorptance at 273 nm.
